# Patterns of drinking in Aboriginal and Torres Strait Islander peoples as self-reported on the Grog Survey App: a stratified sample

**DOI:** 10.1186/s12911-019-0879-8

**Published:** 2019-09-05

**Authors:** KS Kylie Lee, James H. Conigrave, Scott Wilson, Jimmy Perry, Noel Hayman, Catherine Zheng, Mustafa Al Ansari, Michael Doyle, Robin Room, Sarah Callinan, Tanya Chikritzhs, Tim Slade, Katherine M. Conigrave

**Affiliations:** 1 Discipline of Addiction Medicine, Indigenous Health and Substance Use, The University of Sydney, Faculty of Medicine and Health, NHMRC Centre of Research Excellence in Indigenous Health and Alcohol, King George V Building 83-117 Missenden Road, Camperdown, New South Wales 2050 Australia; 2 Centre for Alcohol Policy Research, La Trobe University, Bundoora, Victoria 3084 Australia; 3 Aboriginal Drug and Alcohol Council South Australia, 155 Holbrooks Road, Underdale, South Australia 5032 Australia; 4 Southern Queensland Centre of Excellence in Aboriginal and Torres Strait Islander Primary Health Care (Inala Indigenous Health Service), 37 Wirraway Parade, Inala, Queensland 4077 Australia; 5 School of Medicine, Griffith University, Gold Coast Campus, Brisbane, Queensland 4222 Australia; 6 University of Queensland, School of Medicine, Herston Road, Herston, Queensland 4006 Australia; 7 Curtin University, Health Sciences, National Drug Research Institute, 10 Selby St, Shenton Park, Western Australia 6008 Australia; 8 Faculty of Medicine and Health, Matilda Centre for Research in Mental Health and Substance Use, The University of Sydney, Camperdown campus, New South Wales 2050 Australia; 9 Sydney Local Health District, Royal Prince Alfred Hospital, Drug Health Services, King George V Building, 83-117 Missenden Road, Camperdown, New South Wales 2050 Australia

**Keywords:** Aboriginal, Torres Strait Islander, Australia, Alcohol, Consumption, Patterns, Survey

## Abstract

**Background:**

The Grog Survey App is a visual and interactive tablet computer-based survey application. It has been shown to be an accurate and acceptable tool to help Indigenous Australians describe what they drink.

**Methods:**

The Grog Survey App was used to enquire into patterns of drinking in a stratified sample of Indigenous Australians in urban and remote/regional sites during testing of the App. The App asked about the last four drinking occasions in the past 12 months, including preferred alcohol types and containers; and symptoms of alcohol dependence, based on ICD-11 descriptions. Drinking patterns are presented here using medians and interquartile ranges, and the thresholds set out by the Australian National and Health and Medical Research Council guidelines. Patterns of consumption are compared by gender and remoteness, using Wilcoxon rank-sum test to compare medians. Logistic regressions tested whether alcohol types and drinking containers varied by remoteness.

**Results:**

In this stratified sample most people either consumed nothing (21.7%), or consumed quantities which placed them at short- (95.6%) or long-term risk (47.8%) of harms. Drinkers in remote areas were more likely to drink beer, but less likely to drink pre-mixed spirits. ‘Stubbies’ and other beer glasses were popular in urban areas, compared with ‘slabs’ (cases of beer) in remote/regional areas. The use of improvised containers (i.e. empty juice bottles) did not vary by remoteness. Nearly one in six (15%) current drinkers reported experiencing at least two symptoms of alcohol dependence at least monthly. Average drinks per day was the consumption measure most highly correlated with each dependence symptom (*r* = 0.34–0.38).

**Conclusions:**

The App was able to capture a wide range of preferred alcohol types and containers, and demonstrate a diversity in how alcohol is consumed. This detail was captured in a relative brief survey delivered using an interactive and appealing tablet computer-based application.

## Background

Alcohol is consistently reported as a key concern for Aboriginal and Torres Strait Islander (Indigenous) peoples and communities of Australia [1, 2]. As with populations of First Peoples elsewhere [3, 4], Indigenous Australians are a relatively young population, with a median age of 23 versus 38 for other Australians [5]. Given that alcohol use has been found to be the leading cause of disability and death in people aged 15 to 49 [6], a strong public health concern with the drinking patterns of Indigenous Australians is warranted. As with indigenous peoples in Canada and New Zealand, Indigenous Australians are less likely to drink at all compared with their non-Indigenous counterparts, but those who do drink have a higher risk of alcohol-related harms [7–9]. This has been linked to their intergenerational and current experience of discrimination, socioeconomic disadvantage, grief and loss [10, 11].

Household surveys help inform communities and policy-makers about a range of health risk behaviours. However, there is a lack of reliable data on alcohol use in Indigenous Australians [12–14] where consumption has often been substantially underestimated [13]. In fact, some authors conclude that the most reliable self-report data on alcohol use behaviours in Indigenous Australians was published in 1994 and was based on urban areas only [12]. In other Indigenous peoples around the world there has been limited research into approaches to accurately collecting consumption data [15].

Several studies, ranging from single communities to national household surveys, have reported on patterns of drinking in Indigenous Australians. Many of these were conducted two to three decades ago [16–19]. Most report that Indigenous Australians are less likely than other Australians to drink alcohol in the past year [7, 16, 17, 19], except for one study of young people [18]. Despite this, most studies found an increased prevalence of risky drinking [7, 16, 17], with estimates varying from 54% of Indigenous Australians nationally [7] to 67.9% of female and 68.9% of male drinkers in remote Northern Territory (NT; an internal territory of Australia) [16]. Beverage preferences varied, with beer [16, 17] being most popular in remote Western Australia (WA; a state of Australia) and the NT, and spirits in WA [19] and nationally [7]. No studies have reported on preferred container types, although consultation suggests that alcohol may be poured into non-standard containers like empty soft drink or juice bottles [14] if no glass is available.

Several authors have commented on the difficulty of obtaining accurate self-report data on alcohol use from Indigenous Australians due to factors such as episodic drinking and sharing of drinks [14] that are not adequately captured by most survey tools. Surveys also often require people to convert their consumption into ‘standard’ drinks [12, 14] (each of 10 g ethanol in Australia) and this kind of mental arithmetic can present a challenge to any population, including those who may not have comfort with numbers.

To address these difficulties in collecting self-report alcohol use data (12, 14), we developed the Grog Survey Application [20] (‘App’; ‘grog’ is a commonly used word in Australia for alcohol). It is designed to be an easy-to-use tool to help Indigenous Australians describe what they drink [6]. The App has been shown from previous studies to be accurate when reporting on alcohol consumption [21]. Compared to a clinical assessment using a retrospective diary (conducted by an Aboriginal health professional), the App is able to detect 93% of those who were found to be at short-term risk of harms (specificity: 70%) [21]. The interactive elements of the App allow the participant to choose the type and brand of alcohol they drink, choose the container they drink from, and to drag a slider up and down to demonstrate how full the container was with alcohol [22]. The aim of this paper is to describe the patterns of drinking (including preferred alcohol types and containers, and symptoms of dependence) reported by a stratified sample of Aboriginal and Torres Strait Islander peoples in urban and remote/regional sites during testing of the Grog Survey App.

## Methods

Study methods were designed by investigators in consultation with the Aboriginal Drug and Alcohol Council of South Australia; the Aboriginal Health Council of South Australia (AHCSA), the peak body for Aboriginal community controlled health services in South Australia; and the Aboriginal Drug and Alcohol Network, representing Aboriginal alcohol and other drug workers in New South Wales. Ethical approval was obtained from AHCSA (#04–15-621) and from Metro South Health Human Research Ethics Committee in Queensland (#HREC/16/QPAH/293).

### Recruitment

As recruitment was designed to test the validity of the App with a range of drinkers, and for both genders, stratified convenience sampling was used. Recruitment has been described elsewhere [21]. We aimed to recruit: 20 non-drinkers, 40 non-dependent drinkers and 40 dependent drinkers in each of two Australian states. Aboriginal field research assistants who knew the local community approached potential participants based on observation/anecdotal knowledge of the drinking category that person would be in, and invited them to participate. The research assistants then set up participants on iPads to complete the survey App. Then at the end of each day all iPads were synchronised to the University of Sydney encrypted server, and the App dashboard provided an update of the number of people in each drinking, age and gender category based on participants’ responses to the App survey. This was then used to inform the next day’s recruitment efforts. Participants were classified as non-drinkers by the survey App if they reported not consuming alcohol in the preceding 12 months. To assess presence of alcohol dependence, drinkers were asked on the survey App to rate the frequency of occurrence of each of three dependence criteria (ICD-11) [20, 23]. For the purpose of sampling (only), any participant who responded that one or more of these dependence symptoms occurred at any time in the past 12 months was considered dependent on alcohol.

In urban Queensland (Qld), recruitment was based in an Indigenous primary health care service and surrounding community (August to November 2016). In South Australia (SA), recruitment centred on a regional ACCHS and a remote Aboriginal community controlled drug and alcohol ‘day centre’ (a drop-in service; two periods in August to September 2016 and April to May 2017). Individuals were eligible for inclusion if they self-identified as being Aboriginal and/or Torres Strait Islander and were 16 years or older. Exclusion criteria included obvious intoxication. Participants were reimbursed for their time with a store voucher.

### Data collection

#### Grog survey app

Development of the App and the composition of its survey items have been described elsewhere [6]. Broadly, the App features questions on demographics, alcohol consumption (10 items), alcohol dependence (3 items based on ICD-11 [23]), harms to self or others, treatment access and participants’ feedback on using the App. The App ‘reads out’ the questions in English or in Pitjantjatjara (an Aboriginal language spoken in a region that intersects South Australia, Western Australia and the Northern Territory). The App was designed to take no longer than 20 min to complete.

**Fig. 1:**
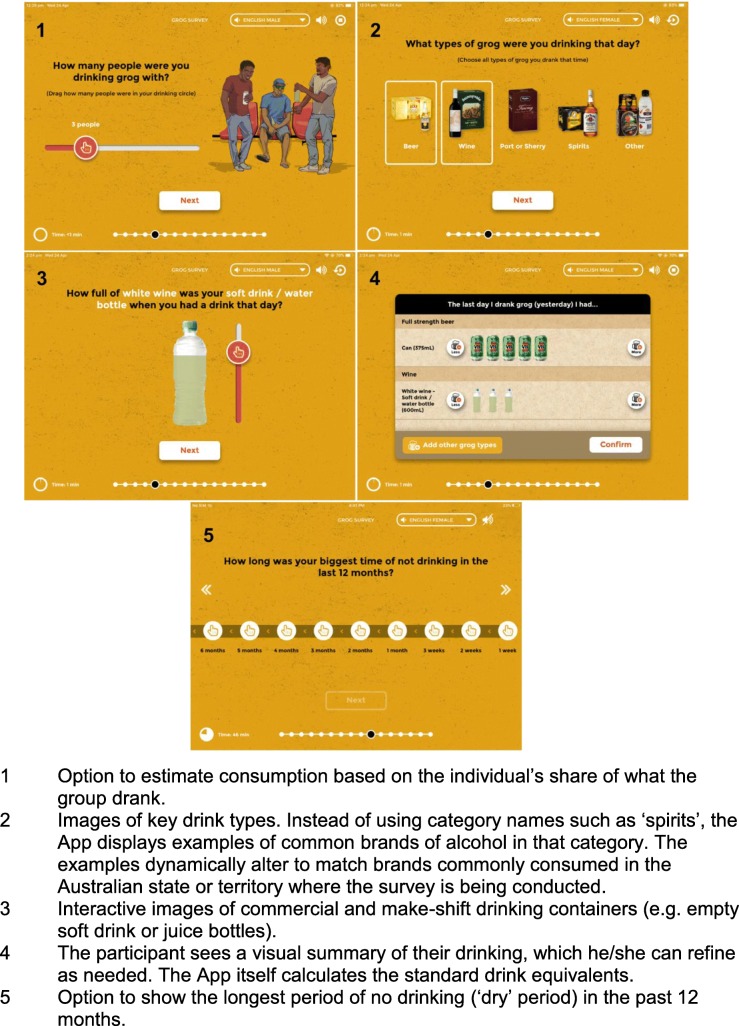
Screengrabs showing key features of the Grog Survey App

The App includes: culturally appropriate questioning style and gender-specific voice and images; ‘translation’ to colloquial English and (for audio) to the local Indigenous language (Pitjantjatjara); interactive visual approaches to estimate quantity of drinking; images of specific brands of alcohol, rather than abstract description of alcohol types (e.g. ‘spirits’); images of commercial and make-shift drinking containers (e.g. empty soft drink bottles); an option to estimate consumption based on the individual’s share of what the group drank; key time points to explain time references for communities that do not routinely use calendars (e.g. ‘since last New Year’ to help explain ‘in the past 12 months’; see Fig. 1).

#### Alcohol consumption

Using an adaptation of the last four occasions (Finnish) method [24] to assess drinking, the App asks respondents to show the date of their four most recent drinking occasions in the past 12 months. Participants are then asked how much alcohol they consumed on each of these occasions. Participants selected pictures of the type of alcohol, the container they drank it from, and how full the container was with alcohol [6]. The App also allowed participants to describe how much alcohol they drank as part of a group, if that was easier for them, and to then show their share. Australian standard drinks (10 g pure alcohol) were calculated by the App itself. For each participant the App calculated the average number of standard drinks consumed per month, per day and per drinking occasion.

The App has been found to be as good or better at identifying risky drinking compared with a clinical interview by an Aboriginal health professional [21]. Average consumption was well correlated in test-retest reliability (r_s_ = .81, *n* = 181, all participants; r_s_ = .81, *n* = 147, drinkers only). In this testing in regional/remote South Australia and urban Queensland, the App was also found to be highly acceptable [22].

#### Three symptoms of alcohol dependence based on ICD-11 criteria

Participants were asked about: 1) Alcohol withdrawal tremors – “*Some people’s hands shake when they stop drinking or before their first drink of the day. How often does this happen to you?”*; 2) Loss of control – *“Some people feel like grog is the boss of them. How often do you feel grog makes all the decisions (so you could not stop drinking, even if you tried)?”*; and 3) Prioritising alcohol over other things – *“Some people spend more time drinking grog than doing other things they need to do, like looking after family, culture or work. How often does this happen with you?”.* Responses for each item were indicated on a five point Likert scale ranging from ‘never’ to ‘most days or every day’. Participants were considered to be dependent if they experienced two or more symptoms at least once per month.

### Hypotheses

We expected that drinking patterns, and container usage would vary by remoteness. Specifically, we anticipated that individuals in remote communities would be more likely to use non-standard drinking containers.

### Analysis

All analyses were performed in R [25]. Medians and interquartile ranges were used to describe drinking patterns due to the skewed nature of consumption data. Participants were classed as being at short-term risk of injury from alcohol if they drank more than four standard drinks on a single occasion, and at long-term risk of chronic diseases if they consumed an average of more than two standard drinks per day [26]. Drinking patterns were calculated separately for males and females and urban and regional/remote dwelling participants. Medians were compared with the Wilcoxon rank-sum test. Logistic regressions were used to test whether different alcohol types and different drinking containers were used by urban and regional/remote participants. Each participant was given a binary classification for having consumed each type of alcohol, or for having used each type of container. These variables became the outcome in a logistic regression where remoteness was the predictor.

## Results

### Participants

Participants were 263 Indigenous Australians (44.1% female) from four communities (1 urban Queensland; 3 remote/regional South Australia). Participants had a mean age of 38.7 (*SD* = 13.9) years. Just under half of participants had completed high school beyond Year 10 (*n* = 121, 46.0%). The majority of participants were unemployed (*n* = 165, 62.7%). Employment did not significantly vary by remoteness (chi-square = 0.20, df = 1, *p* = 0.65). However, more males (42.9%) were employed, than females (30.2%; chi-square = 3.8, df = 1, *p* = 0.05).

### Patterns of drinking

In keeping with the stratified recruitment, nearly eight in ten participants (*n* = 206, 78.3%) said that they drank alcohol in the previous 12 months (84.4% male, 70.7% female; Table 1). As planned with sampling, drinking status did not significantly vary by remoteness (chi-square = 2.23, df = 1, *p* = 0.14). However, despite stratification for gender, men were more likely to be classified as drinkers than women (chi-square = 6.35, df = 1, *p* = 0.01).

**Table 1:** Participant characteristics by drinking status as collected on the Grog Survey App (*n* = 263)

Variable	Non-drinker	Current drinker^a^
Age (mean)	44.2	37.1
Female (%)	59.6	39.8
Employed (%)	12.3	44.2
Remote/regional (%)	68.4	56.3
Urban (%)	43.7	31.6

Note: ^a^ any alcohol at all in the past 12 months. The denominator for the two columns is, respectively, the total number of non-drinkers (*n* = 57) and current drinkers (*n* = 206)

The majority of drinkers in this stratified sample consumed at levels which placed them at short- (95.6%) or long-term risk (47.8%) of harms from drinking. Only a small number of drinkers reported drinking below the recommended national guidelines (*n* = 9, 4.3%). Among drinking participants, drinking occasions were relatively infrequent, with a median of 3.5 drinking occasions per month (*IQR* = 4.7). However, the median number of standard drinks per occasion was high, at 17.3 (*IQR* = 19.5). When drinking is averaged over the month, participants consumed a median of 1.6 (*IQR* = 4.2) standard drinks per day. The median drinking group size was four drinkers (*IQR* = 4.0). Most drinkers (79.6%) reported having had a ‘dry patch’ (i.e. an extended period of time where they did not drink in the past 12 months). Median dry patch duration was 60 days (*IQR* = 128.5).

Drinking patterns tended to vary by gender (see Table 2). Compared to females, males tended to drink more frequently, consume greater quantities of alcohol and have shorter dry patches.

**Table 2:** Drinking patterns by gender as collected on the Grog Survey App (n = 206 drinkers)

Variable	Males median (IQR)	Females median (IQR)	P
Drinking occasions per month	3.5 (4.3)	1.8 (4.3)	< 0.01*
Standard drinks per day	2.2 (4.9)	0.8 (2.5)	< 0.01*
Standard drinks per occasion	19.4 (24.5)	14.8 (16.8)	< 0.01*
Group drinking size	5 (5)	4 (4)	0.06
Dry patch duration (days)	30 (83.5)	90 (171.2)	0.04*

Note: * *p* <  0.05; *p* values calculated from Wilcoxon rank sum test

In this sample, drinking patterns did not vary by remoteness. Wilcoxon rank sums tests showed that the number of drinking occasions per month, average standard drinks per day, standard drinks consumed per drinking occasion, and group drinking size were similar for participants in urban and in remote/regional settings (all *p* > 0.15; males and females combined; results by remoteness are not shown).

### Drinking types and containers

Information on the exact containers used was available for 188 (91.3%) current drinkers. A series of logistic regressions were conducted to better describe whether remote/regional drinkers were more or less likely to report certain types of alcohol and drinking containers. While remote drinkers were more likely to drink beer, they were less likely to drink pre-mixed spirits (Table 3).

**Table 3:** Percentage of drinkers who consumed various types of alcohol, and the odds of those beverages being consumed in remote/regional and urban areas (total *n* = 188 drinkers; logistic regression)

Beverage type	Percent who consumed this	Remote/Regional OR (95% CI)	Urban OR (95% CI)	*p*
Beer	60.7	2.64 (1.49, 4.72)	0.38 (0.21, 0.67)	< 0.01*
Pre-mix	46.6	0.48 (0.28, 0.85)	2.06 (1.18, 3.63)	0.01*
Spirits	19.9	0.61 (0.30, 1.20)	1.65 (0.83, 3.31)	0.15
Mixed drinks	18.0	0.69 (0.33, 1.40)	1.46 (0.71, 2.99)	0.3
Wine	11.7	0.62 (0.26, 1.46)	1.61 (0.68, 3.86)	0.27
Port	5.3	3.70 (0.92, 24.69)	0.27 (0.04, 1.08)	0.1
Cider	4.9	1.86 (0.50, 8.83)	0.54 (0.11, 1.99)	0.38
Cocktail	2.9	0.15 (0.01, 0.94)	6.76 (1.07, 130.87)	0.08

Note: remote/regional or urban OR = the odds of a beverage type being consumed by a person living in a remote/regional or urban community; p = logistic regression *p* value; * *p* < 0.05

‘Stubbies’ and other beer glasses tended to be used more often in urban areas. In contrast, ‘slabs’ (cases of beer) were used more in remote/regional areas. The use of improvised containers (i.e. an empty juice or soft drink bottles) did not vary by remoteness (Table 4).

**Table 4:** Most popular drinking containers and the odds of them being used in remote/regional and urban areas (n=188 drinkers; logistic regression)

Container	Percentage who used it	Remote/Regional OR (95% CI)	Urban OR (95% CI)	*p*
Multi pack	40.8	0.65 (0.37, 1.14)	1.54 (0.88, 2.71)	0.13
Can	34.0	1.81 (1.00, 3.32)	0.55 (0.30, 1.00)	0.05
Stubby	19.4	0.44 (0.21, 0.88)	2.27 (1.13, 4.67)	0.02*
Drinking glass	18.0	0.53 (0.25, 1.07)	1.90 (0.93, 3.95)	0.08
Slab	13.6	2.62 (1.11, 6.94)	0.38 (0.14, 0.90)	0.04*
Bottle	11.2	1.01 (0.42, 2.48)	0.99 (0.40, 2.37)	0.98
Used bottle	6.3	0.90 (0.29, 2.89)	1.11 (0.35, 3.47)	0.85
Cask	5.8	1.09 (0.34, 3.80)	0.92 (0.26, 2.97)	0.88
Wine glass	4.9	0.77 (0.21, 2.83)	1.31 (0.35, 4.83)	0.68
Beer glass	3.9	0.10 (0.01, 0.59)	9.70 (1.68, 183.08)	0.04*
Jug	3.9	0.45 (0.09, 1.89)	2.22 (0.53, 11.04)	0.29
Paper cup	2.9	4.01 (0.63, 77.55)	0.25 (0.01, 1.58)	0.21
Longneck	2.4	0.19 (0.01, 1.29)	5.35 (0.77, 105.64)	0.14
Cocktail glass	1.9	0.77 (0.09, 6.54)	1.30 (0.15, 10.97)	0.80

Note: remote/regional or urban OR = the odds of each container being used by a person living in a remote/regional or urban community; p = logistic regression p.value; * p < 0.05; container volumes are as follows

*Volume of containers in mL: multi pack: 1100 - 3750; can: 375 - 375; stubby (i.e. glass of beer): 275 - 375; drinking glass: 240 - 350; slab (i.e. case of beer): 8280 - 11250; bottle: 500 - 750; used bottle: 500 - 2000; cask: 2000 - 5000; wine glass: 150 - 200; beer glass: 285 - 450; jug: 1140 - 1140; paper cup: 235 - 235; longneck: 660 - 750; cocktail glass: 180 - 180

### Symptoms of alcohol dependence

Nearly one in six (15%) of current drinkers reported experiencing at least two symptoms of alcohol dependence at least monthly. Table 5 shows correlations between frequency of symptoms of alcohol dependence, and each of: consumption patterns, gender and remoteness. The consumption characteristic which correlated the most highly with each of the dependence symptoms was average drinks per day (*r* = 0.34–0.38), however, other drinking indicators (drinking frequency and drinks per occasion) also each correlated significantly with each symptom of dependence. There was a strong correlation between each of the three dependence symptoms (*r* = 0.57–0.61).

**Table 5:** Spearman intercorrelations of symptoms of alcohol dependence and alcohol consumption characteristics, gender and demographics in urban and remote/regional settings for the Grog Survey App in a stratified sample (*n* = 206 drinkers)

	1	2	3	4	5	6	7
1. Loss of control	–						
2. Time spent	0.55*	–					
3. Tremor	0.61*	0.57*	–				
4. Drinking frequency	0.32*	0.31*	0.28*	–			
5. Drinks per occasion	0.23*	0.26*	0.30*	0.18*	–		
6. Average drinks per day	0.35*	0.34*	0.38*	0.81*	0.68*	–	
7. Male	0.07	0.13	0.21*	0.21*	0.21*	0.30*	–
8. Remote	0.18*	0.12	0.19*	0.10	0.06	0.10	0.16*

Note: * *p* < 0.05; Loss of control = diminished control over drinking; Time spent = spending too much time drinking; Tremor = withdrawal tremor; Drinking frequency = number of drinking occasions per month; Drinks per occasion = number of standard drinks per drinking occasion; Average drinks per day = average number of standard drinks per day; Male = binary, participant is male; Remote = binary, participant is from a remote or regional area; Male and Remote were coded as dummy variables, to find the values for females, and urban areas, these correlations can be multiplied by −1

## Discussion

This study is unique in that it presents detailed examples of alcohol use behaviours in Indigenous Australians from two states, and from both urban and regional/remote settings, using a newly designed, tablet computer-based survey tool. Unlike national household surveys [2, 7], this App allows collection of data on preferred drinking containers (including non-standard containers that alcohol is poured into) and on symptoms of dependence. While past studies of drinking patterns for Indigenous Australians in single states or territories have been conducted [16–19], none report on use of non-standard containers or length of dry periods reported by current drinkers. By observation, and from this data, these are important to consider. The tablet computer-based survey (Grog Survey App) has been found to be an accurate [21] and acceptable [22] tool to collect detailed self-report data on alcohol use behaviours in this population.

### Patterns of drinking in this stratified sample

This sample was stratified as it was part of a study designed to test the App’s performance across a range of drinkers. The results on drinking patterns are valuable as an illustration of the range of drinking patterns, and the ability of this survey tool to capture them. However, they should not be mistaken for prevalence data.

Relative to national averages, this sample’s participants tended to be older, and it included more males, and more unemployed individuals. By contrast, in the 2016 Australian census, 50.4% of Indigenous people are female, the median age is 23 and 18.2% are unemployed [27]. More than a third (37.4%) of drinkers in the current sample were living in an area where there were some restrictions to alcohol access, so even among the participants from remote settings, patterns of drinking may differ from other remote regions.

We set out to recruit different types of drinkers but were unable to recruit many drinkers who consumed within recommended national guidelines (just 4.3% of drinkers were drinking within national guidelines). This could be because lighter drinkers were not known by Aboriginal field research assistants to be drinkers, and so were less likely to be recruited with this convenience stratified sampling. It also could have occurred if low risk drinking was relatively uncommon in the relatively low socio-economic regions where recruitment took place. By comparison, just over one in eight (13%) urban Indigenous Australians in a national urban household survey were low-risk drinkers in a 1994 survey, which is reportedly one of the most accurate available [2, 12]. That survey used a higher threshold for low risk drinking, consistent with the current national guidelines of the time, of no more than four or less standard drinks per day for men [2].

Episodic drinking was common in this sample, with relatively low frequency of consumption (3.5 median drinking days in the last month for men, 1.8 for women). However, when people did drink, they reported a high median number of standard drinks per occasion (19.4 for men, 14.8 for women). These figures are plausible, as a similar heavy episodic pattern of drinking was seen in the 1994 national survey of urban Indigenous Australians (aged 14+), in which the commonest category of consumption was 13+ standard drinks per occasion (42% men, 21% women) [2]. The typical frequency of drinking was at least once a week [2]. More recent national surveys do not report on the actual quantity consumed on single occasions of drinking [28, 29]. Instead data are presented as a proportion of people who consume more then four standard drinks in a drinking session (the current NHMRC drinking threshold for short-term risk of injury from alcohol).

Pattern of drinking to intoxication is also seen in the developing world, where drinkers tend to be polarised into non-drinkers and risky drinkers, with few low-risk drinkers [30]. Studies of the British Public Service also suggest that individuals who are higher up the power hierarchy and therefore are likely to have a greater sense of control over their lives are more likely to drink at moderate levels [31].

Drinkers in this sample sometimes had protracted dry periods between drinking days (median of 30 days for men and 90 days for women). We expected that drinkers from remote communities would have longer dry periods than urban drinkers, particularly as some remote participants came from regions where access to alcohol is restricted. However no difference was found in dry period length based on remoteness (urban versus remote/regional). This finding is consistent with studies [17, 32] that show that some Indigenous individuals drink only for special occasions (e.g. weddings, funerals, football grand finals). We were unable to find other studies of Australia’s First Peoples that report on the length of periods when current drinkers abstain from drinking. Previous studies have focused only on dependent drinkers who try to quit and the duration of abstinence before relapse [17, 33]. However, in our sample excluding dependent drinkers did not significantly change the median length of dry periods (data not shown).

Dry periods, such as those reported in this study, would be difficult to capture in commonly used survey and screening tools. For example the screening tool AUDIT-C (Alcohol Use Disorders Identification Test-Consumption), which is recommended for use in Aboriginal community controlled health services in Australia, would not capture this information as it assumes regular patterns of drinking. For example, AUDIT-C question 1 asks: “How often do you have a drink containing alcohol?” (with response categories as a Likert scale from: never to most days/every day).The Australian Bureau of Statistics’ National Health Survey uses a seven day diary method [34]. Our data suggests that asking about the past week is not a long enough period to describe drinking patterns in this population. While AUDIT-C can capture longer time periods [35], regular patterns of drinking cannot be assumed in this population [20]. Staff administering AUDIT-C in clinical settings may need guidance on how to code responses from drinkers who have lengthy dry periods, or recent changes to drinking patterns.

As our sample was stratified by drinking levels it is not suprising that we did not find differences in quantity consumed between urban and regional/remote sites. National surveys of Indigenous Australians report that individuals from non-remote areas (55%) are significantly more likely to consume alcohol at levels which place them at short-term risk of harms from drinking than those in remote areas (48%) [7]. This may be in part due to the differing access of Indigenous communities across Australia to alcohol.

#### Preferred alcohol types and containers

Among urban and remote drinkers, beer (60.7%), premix (46.6%) and spirits (19.9%) were consumed by most drinkers. While urban drinkers were twice as likely to report having consumed premixes, remote/regional drinkers were 2.6 times likely to report beer. These findings reinforce the challenge of supply control measures that target only one alcohol type (e.g spirits or cask wine). Some studies suggest that Indigenous Australians who drink (as with other drinkers) may switch their preferred beverage type to access the cheapest alcohol [36, 37]. This does not appear to be the case in this stratified sample, however, as wine (including cask wine – often the cheapest form of alcohol in Australia), was consumed by just 11.7% of drinkers from both urban and remote/regional sites.

This is the first study to report on preferred drinking containers, including non-standard containers such as empty soft drink or juice bottles or a metal mug (pannikin) that alcohol is poured into. The interactive tablet computer interface allowed collection of such detail. Data from this sample suggests a diversity in container preference between the urban and remote/regional sites. We had expected remote drinkers, who are often drinking in bush or parkland, to be more likely to use improvised containers. However, improvised containers were used just as commonly in urban settings. This may reflect both the culture of sharing, and relative socioeconomic disadvantage of the recruitment sites.

A broad pattern emerged where remote/regional drinkers were more likely to consume from containers in which alcohol is typically sold in bulk (i.e. cans of beer sold in a case) and less likely to drink from single containers commonly available from licensed venues (i.e. beer glasses, including ‘stubbies’). This may be due to travel by remote drinkers to access alcohol if they live in a community with alcohol restrictions. If so, they may purchase alcohol in bulk [38]. Also, in some regional or remote towns, Aboriginal individuals may not feel welcome to drink in licensed venues, or may simply prefer to drink in groups outdoors under a tree (personal communication and observation). Single containers (e.g. can of beer) may also be relatively more expensive and so out of financial reach, as they are typically purchased from licensed venues (like bars, restaurants).

#### Symptoms of alcohol dependence

The App survey included a screen for dependence based on an operationalisation of the three key features of alcohol dependence (ICD-11) [23]. Dependence questions were asked on the App in plain English language and checked with Aboriginal health profesionals with urban and remote experience. Despite the high per occasion consumption, and over-sampling of drinkers with presumed dependence (40% of the stratified sample), just three in 10 individuals reported one or more symptoms of dependence on the App. This may be because the low frequency and stop-start drinking pattern seen in this sample reduces the risk of tolerance and hence of dependence. In keeping with this, one study from Queensland reported an absence of withdrawal syndromes when alcohol supply was stopped in a remote Aboriginal and Torres Strait Islander community [39]. The high per occasion consumption without a high prevalence of dependence has similarities to other populations which episodically drink to intoxication such as university students (where ≥10 or ≥ 15 standard drinks per occasion are regularly reported) [40]. A study from remote Western Australia found that (as would be expected), Aboriginal people who were ‘constant’ drinkers reported higher scores on the CAGE screening tool (suggesting likely dependence) than did ‘episodic’ drinkers [17].

Concerns have been expressed about the ability of researchers or clinicians to measure dependence across cultures, including how readily certain concepts such as salience or loss of control translate across cultures [15]. However the strong intercorrelation of the three dependence measures in this study provides some evidence towards their utility in this stratified sample of Australian Indigenous peoples. For example, if someone reported tremor when they stopped drinking, they also reported loss of control (*r* = 0.61), or prioritising alcohol over other things (*r* = 0.57). Similarly, as expected, the average number of drinks per day was also modestly correlated with symptoms of dependence (*r* = 0.34–0.38). Further validation study, and data on the prevalence of dependence would be valuable to assist with service planning, particularly given the reports of shortage of detoxification and residential rehabilitation beds for Indigenous communities [41].

### Limitations

As this study used stratified sampling, these results do not provide estimates of prevalence but rather examples of a range of drinking patterns. The higher unemployment rate found in this sample may have been associated with higher alcohol consumption figures consumption [42]. There was no control group of non-Indigenous participants against which results could be compared. No validation study was conducted on dependence criteria. This was because a diagnostic interview for dependence was not possible because of lack of availability of addiction psychologists or addiction physicians with understanding of the local Indigenous culture. Future research could look to interview a whole community (with consent) and compare consumption data as self-reported on the App against sales data, and explore associations of particular drinking patterns using data linkage analyses with hospital or other data. We are unable to provide the raw participant-level data used in the reported analyses. This is because data was collected from small Australian Aboriginal communities and we do not have ethical clearance to release these datasets.

## Conclusion

A stop-start pattern of drinking with heavy per occasion consumption was common in this stratified sample of urban, regional and remote Indigenous drinkers. The wide range of preferred alcohol types and containers suggests a diversity in how alcohol is consumed, including differences by remoteness. These survey results demonstrate the depth of detail that can be collected using a visual, interactive and appealing tablet computer-based format. This tablet computer-based App was able to reach a population otherwise difficult to observe by conventional research strategies. Such technology offers new potential to understand drinking patterns in populations that can be hard to reach by pen-and-paper or face-to-face surveys.

## Data Availability

Data for this project is stored at the University of Sydney based at Drug Health Service, KGV Building, Missenden Road, Camperdown New South Wales 2050 Australia. We are unable to provide the raw participant-level data used in the reported analyses. Data was collected from small Australian Aboriginal communities and we do not have ethical clearance to release these datasets.
